# Physiological and Immuno-Antioxidant Responses of *Mercenaria mercenaria* to Salinity Stress

**DOI:** 10.3390/biology15181623

**Published:** 2026-09-15

**Authors:** Gaofu Chen, Tinglong Hou, Peng Wang, Chenhui Li, Hui Wang, Shuang Liang, Jian Liang, Zhixiong Zhou, Xiaoyu Wang, Limei Chen, Yongren Li, Yongjun Guo, Huiru Liu

**Affiliations:** 1 Key Laboratory of Smart Breeding (Co-Construction by Ministry and Province, Ministry of Agriculture and Rural Affairs), Tianjin Agricultural University, Tianjin 300384, China; 2 Tianjin Key Laboratory of Aqua-Ecology and Aquaculture, College of Fisheries, Tianjin Agricultural University, Tianjin 300384, China; 3 Engineering Research Center of Tropical and Subtropical Aquatic Ecological Engineering, Ministry of Education, Key Laboratory of Aquatic Eutrophication and Red Tide Prevention of Guangdong Higher Education Institutes, Institute of Hydrobiology, Jinan University, West 601 Huangpu Avenue, Tianhe District, Guangzhou 510632, China

**Keywords:** *Mercenaria mercenaria*, salinity stress, survival, gill morphology, enzyme activity

## Abstract

This study evaluated the effects of salinity stress on survival, gill morphology, and immune-, osmoregulatory-, and antioxidant-related enzyme activities in *Mercenaria mercenaria*. Nine salinities (0–50, practical salinity units, PSU) were tested, with 30 as the control. Survival was monitored for 168 h, and median lethal salinities under hypo- and hypersaline conditions were estimated. Gill histology was examined at 24, 72, and 168 h under salinities 15, 30, and 50, while ACP, AKP, SOD, CAT, and Na+/K+-ATPase activities were measured at five time points. *M. mercenaria* showed substantial tolerance to salinity stress. After 168 h, cumulative mortality was 20% at salinity 45 and 22% at salinity 20, with median lethal salinities of 52.232 and 15.097, respectively. Both low and high salinity caused gill damage, including filament shrinkage at 24 h and wider inter-filament spaces after 72 h. Enzyme activities generally increased initially, then declined and stabilized, although response timing differed among enzymes. These results indicate strong early phase physiological plasticity (<72 h), whereas prolonged stress (>72 h), which could increase mortality risk, may reflect either impaired osmoregulation, antioxidant capacity and immune defense, or the establishment of physiological acclimation. The findings support pond culture management and breeding of salinity-resilient strains.

## Simple Summary

Salinity, or the amount of salt in water, can change considerably in coastal and pond environments and may affect the survival and health of hard clams. This study examined how the hard clam, *Mercenaria mercenaria*, responds to both low and high salinity. We assessed survival, changes in gill structure, and the activity of enzymes involved in stress resistance, body salt balance, and immune protection. The results showed that hard clams can tolerate a relatively wide range of salinities, although prolonged exposure to very low or high salinity increased mortality. Changes in gill structure were observed under both low- and high-salinity conditions. The clams also showed rapid physiological responses during the first 72 h, with several protective enzyme activities increasing before later declining or stabilizing. These findings suggest that hard clams can adjust to short-term changes in salinity, but prolonged extreme salinity may weaken their ability to maintain normal body functions or require longer-term adjustment. The results may help improve salinity management in hard clam farming and support the breeding of strains that are more tolerant to changing environmental conditions.

## 1. Introduction

*Mercenaria mercenaria*, also known as the northern quahog, is a commercially important marine bivalve with substantial ecological and aquaculture value [1]. Native to the Atlantic coast of North America, it is distributed from Nova Scotia to Florida and supports both aquaculture and wild fisheries [2]. In China, *M. mercenaria* has become an important pond-cultured shellfish species in coastal farming areas because of its high market value and tolerance to varying salinity conditions [3]. Recent studies have further highlighted its potential as a target species for genetic improvement and environmental adaptation research, underscoring its importance in modern shellfish aquaculture [4].

Salinity is one of the most important environmental factors governing the distribution, physiology, and production performance of coastal aquaculture species [5]. In estuarine and nearshore systems, abrupt salinity fluctuations can result from extreme rainfall, drought, storm surges, and altered freshwater inflow, and these events are increasingly recognized as major threats to shellfish aquaculture [6]. In hard clam aquaculture, this issue is particularly relevant because production is typically conducted in relatively high-salinity waters and can be severely affected by prolonged hyposaline episodes following heavy precipitation [7]. Although hard clams are generally regarded as salinity tolerant, recent studies indicate that this tolerance has clear limits and that both hypo- and hyper-salinity can impair survival, cellular homeostasis, and physiological performance [3,8]. More broadly, studies of marine osmo-conformers have shown that salinity stress disrupts intracellular osmolyte balance, redox homeostasis, and energy metabolism, thereby increasing the physiological costs of acclimation [9].

Among bivalve organs, the gills are central to the response to salinity stress because they are directly exposed to the external environment and perform gas exchange, ion transport, and filter-feeding functions [10]. Consequently, gill tissues are often among the earliest sites of structural and functional damage under salinity stress [11]. Recent studies in bivalves have shown that salinity fluctuations can induce gill cell swelling, ciliary damage, vacuolation, apoptosis, and marked changes in the activities of immune- and antioxidant-related enzymes [12]. In parallel, salinity stress can activate antioxidant defenses and non-specific immune responses, reflecting the close coupling among osmotic adjustment, oxidative balance, and physiological defense [13]. These patterns have been documented not only in Crassostrea gigas [14] and *Sinonovacula constricta* [15], but also in hard clams, in which recent metabolomic, biochemical, and transcriptomic studies have revealed salinity-dependent changes in ion transport, amino acid metabolism, oxidative stress, and ammonia detoxification pathways [3,16].

Recent studies on hard clams have provided important insights into salinity adaptation at the molecular and cellular levels. Transcriptomic analyses have revealed that short-term exposure to low salinity differentially regulates genes involved in ion transport, protein processing, and stress signaling [17]. In parallel, prolonged salinity challenge induces significant alterations in hemocyte physiology, lysosomal activity, reactive oxygen species production, phagocytosis, and osmotic status, suggesting coordinated cellular adjustment across salinity regimes [8]. Acute salinity challenge has also been shown to alter hemocyte concentration, immune function, and the expression of solute-carrier and immune-related genes, whereas survival analyses suggest that M. mercenaria may be more tolerant of hypo-salinity than extreme hypersalinity under some experimental conditions [18]. However, most previous studies have focused on isolated biological dimensions, such as hemocyte physiology, specific metabolites, or broad transcriptomic profiles. To date, an integrated evaluation combining whole-organism survival, gill histopathology, osmoregulatory capacity, non-specific immunity, and antioxidant defenses under both hyposalinity and hypersalinity stress remains lacking. To address this gap, the present study provides a multi-level assessment linking structural integrity to physiological responses in *M. mercenaria* [8,17,18].

Against this background, the present study investigated the responses of M. mercenaria to both hypo- and hyper-salinity stress at multiple biological levels. Specifically, we examined survival, gill histomorphology, and the activities of key enzymes associated with osmoregulation [Na+/K+-ATPase (NKA)], nonspecific immunity [acid phosphatase (ACP) and alkaline phosphatase (AKP)], and antioxidant defense [superoxide dismutase (SOD) and catalase (CAT)]. We hypothesized that both hypo- and hyper-salinity stress induce gill tissue damage, osmoregulatory disruption, and differential alterations in immune and antioxidant enzyme profiles in *M. mercenaria*. Furthermore, at equivalent deviations from optimal salinity, hyper-salinity exerts significantly more severe adverse physiological impacts than hyposalinity. By integrating organismal performance, tissue-level injury, and biochemical responses, this study contributes to a more comprehensive understanding of salinity tolerance in hard clams and offers practical support for aquaculture management and strain improvement.

## 2. Materials and Methods

### 2.1. Source of Experimental Animals

All hard clams used in this study were collected from aquaculture ponds operated by Tianjin Hengqian Aquaculture Company (Tianjin, China; pond salinity 30). Healthy individuals of similar size were selected, with a mean body weight of 30.0 ± 6.5 g, shell length of 45.4 ± 3.8 mm, shell width of 25.3 ± 2.9 mm, and shell height of 38.6 ± 3.2 mm. After collection, the clams were cleaned to remove epibionts and surface debris and then acclimated for 7 days in aerated aquaria at salinity 30 ± 1, to allow individuals to recover from sampling-related handling stress. During acclimation, the clams were fed daily with a liquid culture of *Isochrysis galbana* at a feeding ration of 2 × 10^4^ cells/mL. Dead individuals, uneaten feed, and feces were removed promptly, and approximately one-third of seawater was renewed regularly to maintain suitable culture conditions. Water temperature was held constant at 25 ± 1 °C, pH at 8.1, dissolved oxygen > 5.0 mg/L, and a 12 h light:12 h dark photoperiod was applied. Salinity was measured using an optical refractometer.

### 2.2. Survival Assessment

To evaluate survival under salinity stress, nine salinity treatments (0, 5, 10, 15, 20, 30, 40, 45, and 50, practical salinity units, PSU) were established, with salinity 30 serving as the control. A preliminary trial with salinity gradients ranging from 0 to 60 (5-salinity-unit intervals, 30 clams per tank) was conducted to inform the salinity levels selected for the formal stress-exposure experiment. Fifty clams were assigned to each treatment group and maintained in 20 L aquaculture tanks. Individual animals within each tank were used as biological replicates. All other experimental conditions were consistent with Section 2.1. Behavioral responses were monitored throughout the experiment, cumulative mortality was calculated, and the 168-h median lethal salinity (LC_50_) was estimated by Probit regression in SPSS 22.0 without log-transformation of salinity values. The resulting LC_50_ value was then used to inform the selection of salinity levels for subsequent experiments.

### 2.3. Experimental Design

Three days after the end of the survival assessment, based on the results, together with preliminary-trial observations and practical aquaculture considerations, three salinity treatments were selected for the subsequent stress experiment: a hypersaline treatment (salinity 50), a hyposaline treatment (salinity 15), and a control treatment (salinity 30). Healthy individuals of similar size were selected, with a mean body weight of 31.1 ± 4.2 g, shell length of 46.6 ± 2.6 mm, shell width of 26.2 ± 1.8 mm, and shell height of 37.7 ± 4.0 mm. The 7-day experiment was conducted in 20 L aquaculture tanks with three treatments and three replicates per treatment. Each replicate contained 50 clams. Experimental seawater was prepared by mixing artificial sea salt (Sea Water Crystal; Jiangxi Haiding Technology Co., Ltd., Ji’an, China) with freshwater. All other experimental conditions were consistent with Section 2.1. Before the experiment, the hard clams were starved for 24 h and then randomly assigned to tanks at the designated salinities. Clams were not fed during the exposure period to prevent the algae culture (grown at salinity 30) from altering experimental salinities. Furthermore, hard clams typically undergo valve closure and filtration inhibition under acute salinity challenge, making supplemental feeding largely ineffective. Other water-quality parameters were maintained as constant as possible throughout the experiment. The clams were not fed during the exposure period, and approximately one-third of the seawater was renewed daily. Every 6 h (4 times over 24 h), the clams’ behavior and mortality were monitored to remove dead clams. At 24, 48, 72, 120, and 168 h after exposure, three clams were randomly collected from each of the three replicate tanks per treatment for subsequent analyses, totaling nine clams per treatment at each sampling time point.

### 2.4. Sample Collection

The gills and digestive gland were dissected from each hard clam. The gills were fixed in 10% neutral buffered formalin in 50 mL centrifuge tubes at room temperature until histological processing. The digestive gland was immediately transferred to cryogenic tubes, snap-frozen in liquid nitrogen, and stored at −80 °C for subsequent biochemical analyses.

### 2.5. Enzyme Activity Assays

For enzyme activity analysis, 0.1–0.2 g of digestive gland (dissected from the whole visceral mass by removing other accessory tissues) was placed into a 2-mL centrifuge tube containing two to three steel beads. Cold 0.9% saline was then added at a tissue-to-saline ratio of 1:9 (*w*/*v*), and the samples were homogenized using a high-speed tissue grinder (Shanghai Cebo Biotechnology Development Center, Shanghai, China). The homogenates were centrifuged at 3500 rpm for 10 min in a refrigerated centrifuge (Thermo Fisher Scientific, Waltham, MA, USA), and the supernatants were collected for enzyme assays.

Commercial assay kits from Nanjing Jiancheng Bioengineering Institute (Nanjing, China) were used according to the manufacturer’s instructions. SOD activity was measured using the WST-1 method with the SOD Assay Kit (Cat. No. A001-3), and CAT activity was determined using the ammonium molybdate method with the CAT Assay Kit (Cat. No. A007-1). ACP and AKP activities were measured using the ACP Assay Kit (Cat. No. A060-2) and AKP Assay Kit (Cat. No. A059-2), respectively, both based on microplate assays. NKA activity was determined using the NKA Assay Kit (Cat. No. A070-2) with a microplate assay.

### 2.6. Gill Histological Observation

The gills were fixed in 10% neutral buffered formalin, dehydrated through a graded ethanol series, cleared, and embedded in paraffin. Paraffin sections were cut at 5 µm using a microtome and stained with hematoxylin and eosin (H&E) for histological examination. For each treatment at each sampling time point, three clams were randomly selected from each replicate (total of 9 clams per treatment). Each clam was sectioned, and multiple sections and views were observed for each sample. The stained sections were mounted on glass slides with neutral resin and examined under a slide-scanning microscope (DSScanner and Motic DSAssistant Lite, Motic, Xiamen, China). Images were captured and archived for further analysis. Gill histopathological evaluation was performed by purely qualitative microscopic observation.

### 2.7. Statistical Analysis

Statistical analyses were performed using Microsoft Excel 2025, GraphPad Prism 10.1.2, and IBM SPSS Statistics 23.0. The main effects of salinity treatment and time, as well as their interaction, were analyzed using two-way analysis of variance (ANOVA). Differences between the control and stress groups were evaluated using independent samples *t*-tests, and within-group differences among time points were analyzed using one-way ANOVA. The value of *p* < 0.05 was considered statistically significant. Prior to analysis, data were tested for normality and homogeneity of variance.

## 3. Results

### 3.1. Survival of Hard Clams Under Different Salinity Stresses

**Figure 1:**
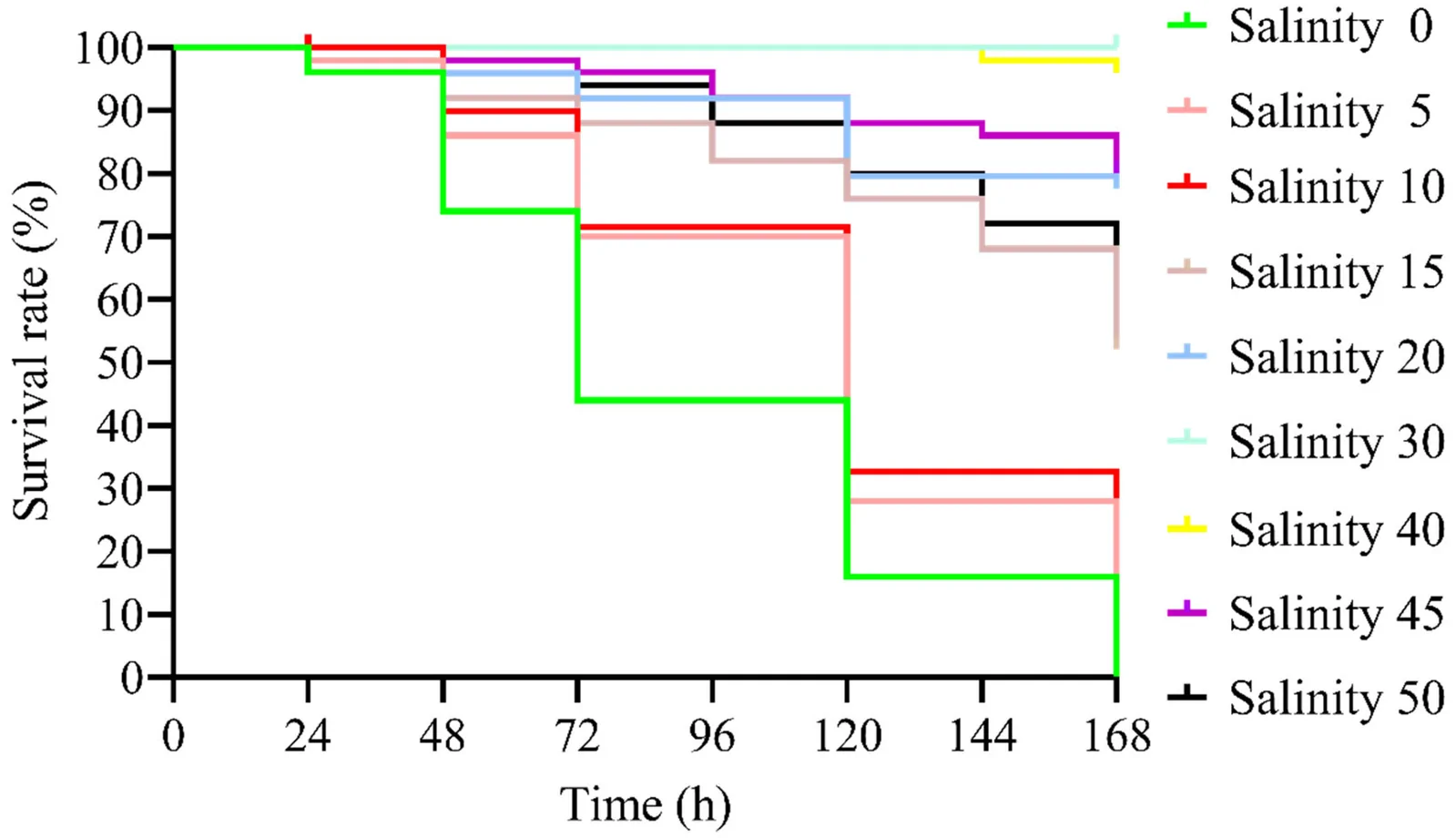
Temporal survival rates of hard shell clams under varying salinity treatments.

The survival of hard clams varied markedly among salinity treatments (Figure 1). Throughout the experiment, clams in the control group (salinity 30) remained in good physiological condition, with fully extended siphons, relaxed adductor muscles, clear and odorless culture water, and 100% survival.

In the salinity 40 group, some individuals exhibited shell closure, but no obvious acute stress response was observed initially. Mortality was first recorded at 144 h, and survival remained 96% at that time. In the salinity 45 group, partial siphon extension was observed, and mortality began to increase at 48 h after exposure; survival declined to 80% by 168 h.

In the salinity 50 group, clams closed their shells completely and became inactive, and the culture water rapidly became turbid. Mortality was first observed at 48 h and increased thereafter, with survival decreasing to 64% by 168 h. A similar pattern was observed in the 15 group, in which mortality began at 24 h and survival declined to 52% by 168 h. Although survival in the salinity 20 group remained 78% at 168 h, mortality also began at 24 h, and the behavioral response resembled that observed in the salinity 45 group. Under extreme low-salinity stress (0, 5, and 10), final survivors numbered 0, 4, and 7 individuals, respectively, to the end of the experiment, and mortality was recorded daily.

Probit regression analysis revealed that the 168-h median lethal salinity (LC50) for M. mercenaria was 15.097 (95% CI: 13.913–16.386) under hyposaline conditions (salinity range 0–30) and 52.232 (95% CI: 49.850–57.519) under hypersaline conditions (salinity range 30–50). Based on the survival data across the salinity gradient and the salinity range commonly used in pond aquaculture, salinities of 50 and 15 were selected for the subsequent salinity-stress experiments.

### 3.2. Gill Histological Changes Under Different Salinity Stresses

**Figure 2:**
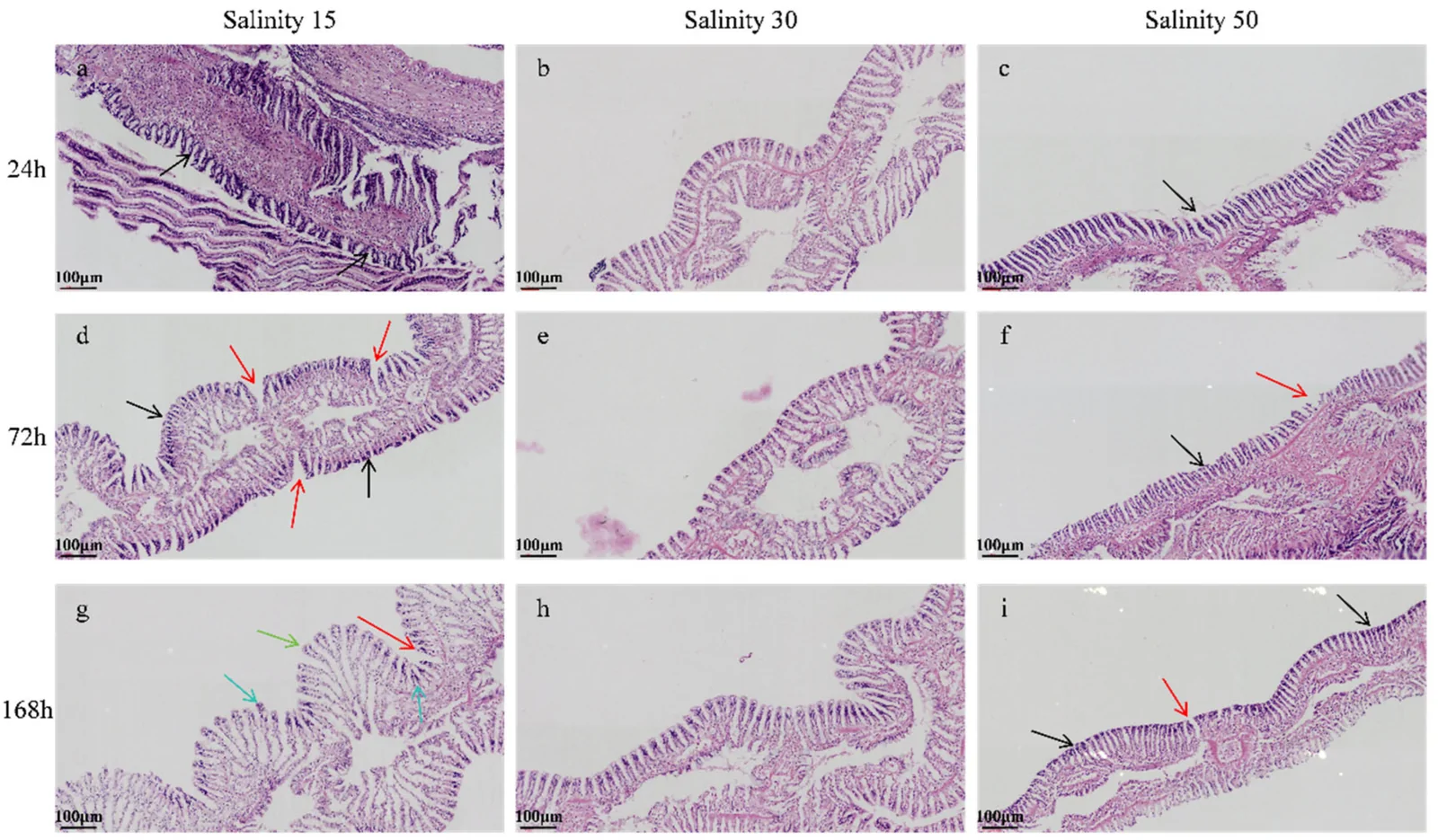
Histopathological assessment of gill tissue in hard shell clams. Panels (**a**,**d**,**g**) correspond to clams exposed to a salinity of 15, panels (**b**,**e**,**h**) represent the control group at a salinity of 30, and panels (**c**,**f**,**i**) show clams at a salinity of 50. Magnification: 200×. Arrows indicate specific cellular alterations: green, cell swelling; blue, nuclear displacement in gill filaments; black, gill filament contraction; red, degeneration of gill filament cells.

The gills are major organs for respiration and filter feeding in hard clams (Figure 2). Histopathological changes in gill tissues were examined by H&E staining after 24, 72, and 168 h of exposure. In the 30 group, the gill structure remained intact and well organized. The gill filaments were regularly arranged, with no obvious degeneration or nuclear displacement.

In the 50 group, gill filaments were shrunken and irregularly arranged at 24 h, and the spacing between adjacent filaments increased. By 72 h, cellular degeneration had become evident in the gill filaments. At 168 h, the filaments were swollen, and the nuclei were displaced from their normal positions.

In the 15 group, gill filaments also showed shrinkage and disorganization at 24 h. After 72 h, degeneration was observed in most gill filament cells. At 168 h, the inter-filament spacing had increased markedly and was accompanied by severe filament shrinkage. These results indicate that both hypersaline and hyposaline stress caused progressive structural damage to gill tissues.

### 3.3. Enzyme Activities in the Digestive Gland Under High-Salinity Stress

**Figure 3:**
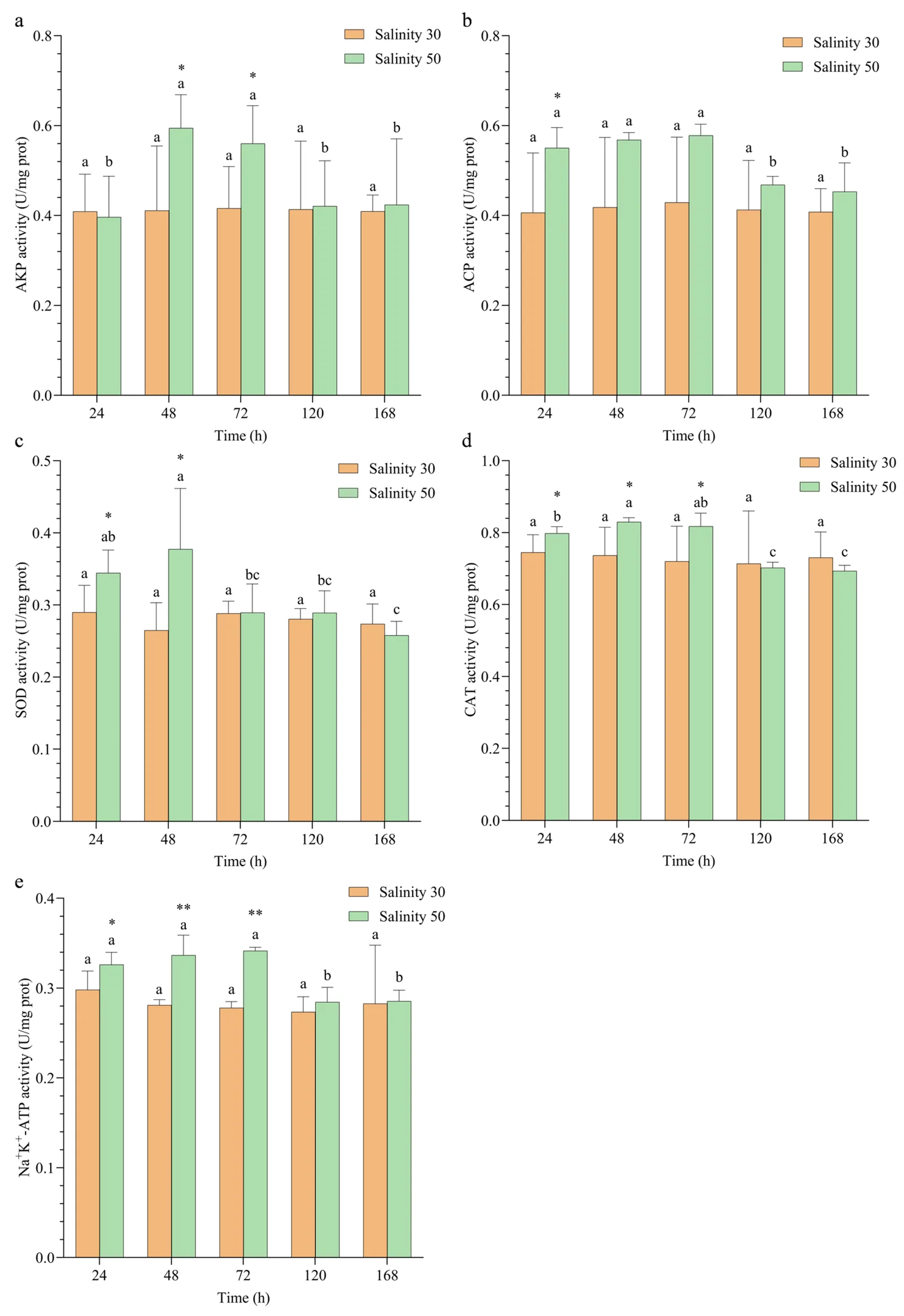
Temporal changes in the activities of immune-related, antioxidant, and osmotic regulation enzymes in the digestive gland of hard shell clams under high-salinity stress. (**a**–**e**) show the activities of AKP, ACP, SOD, CAT and NKA, respectively; * indicates significant difference compared with the control (*p* < 0.05); ** indicates highly significant difference compared with the control (*p* < 0.01). Different lowercase letters indicate significant differences among time points within the same salinity treatment (*p* < 0.05).

As shown in Figure 3, under high-salinity stress (salinity 50), AKP activity showed an initial increase followed by a gradual return to the control level during the experimental period. A peak value of 0.595 ± 0.074 U/mg prot was observed at 48 h, whereas AKP activity in the 30 group remained relatively stable. Compared with the control group, AKP activity did not differ significantly at 24 h, but was significantly elevated at 48 and 72 h (*p* < 0.05), reaching 1.45-fold and 1.35-fold of the control level, respectively. By 120 and 168 h, AKP activity had returned to baseline. High-salinity stress exerted a significant time-dependent effect on AKP activity in the digestive gland (*p* < 0.05).

ACP activity under 50 salinity stress showed a similar temporal pattern, increasing initially and then gradually returning to the control level. ACP activity peaked at 72 h, with a value of 0.578 ± 0.025 U/mg prot, whereas ACP activity in the 30 group remained relatively stable. Compared with the control group, ACP activity was significantly higher at 24 h (*p* < 0.05), reaching 1.36-fold of the control level, and remained elevated at 48 and 72 h. By 120 and 168 h, ACP activity had returned to the control level (*p* > 0.05). High-salinity stress significantly affected ACP activity over time (*p* < 0.05).

SOD activity in the 50 group also exhibited an early increase followed by recovery to the control level. Compared with the control group, SOD activity was significantly higher at 24 and 48 h (*p* < 0.05), reaching 1.13-fold and 1.43-fold of the control level, respectively, with a peak value of 0.377 ± 0.084 U/mg prot at 48 h. As exposure continued, SOD activity gradually returned to baseline, whereas SOD activity in the 30 group remained stable. High-salinity stress had a significant time-dependent effect on SOD activity in the digestive gland (*p* < 0.05).

CAT activity under 50 salinity stress followed the same general pattern. At 24, 48, and 72 h, CAT activity was significantly higher than that in the control group (*p* < 0.05), reaching 1.07-, 1.15-, and 1.13-fold of the control level, respectively, with a peak value of 0.830 ± 0.012 U/mg prot at 48 h. At 120 and 168 h, CAT activity had gradually returned to baseline, and no significant difference from the control group was detected. A significant interaction between time and high-salinity stress was detected for CAT activity in the digestive gland (*p* < 0.05).

NKA activity in the 50 group likewise showed an initial increase followed by recovery to the control level. Compared with the control group, NKA activity was significantly higher at 24 h (*p* < 0.05) and highly significantly elevated at 48 and 72 h (*p* < 0.01), reaching 1.09-, 1.20- and 1.23-fold of the control level, respectively, with a peak value of 0.341 ± 0.004 U/mg prot at 72 h. As exposure time increased, NKA activity gradually returned to baseline. The interaction between time and salinity stress significantly affected NKA activity in the digestive gland (*p* < 0.05).

### 3.4. Enzyme Activities in the Digestive Gland Under Low-Salinity Stress

**Figure 4:**
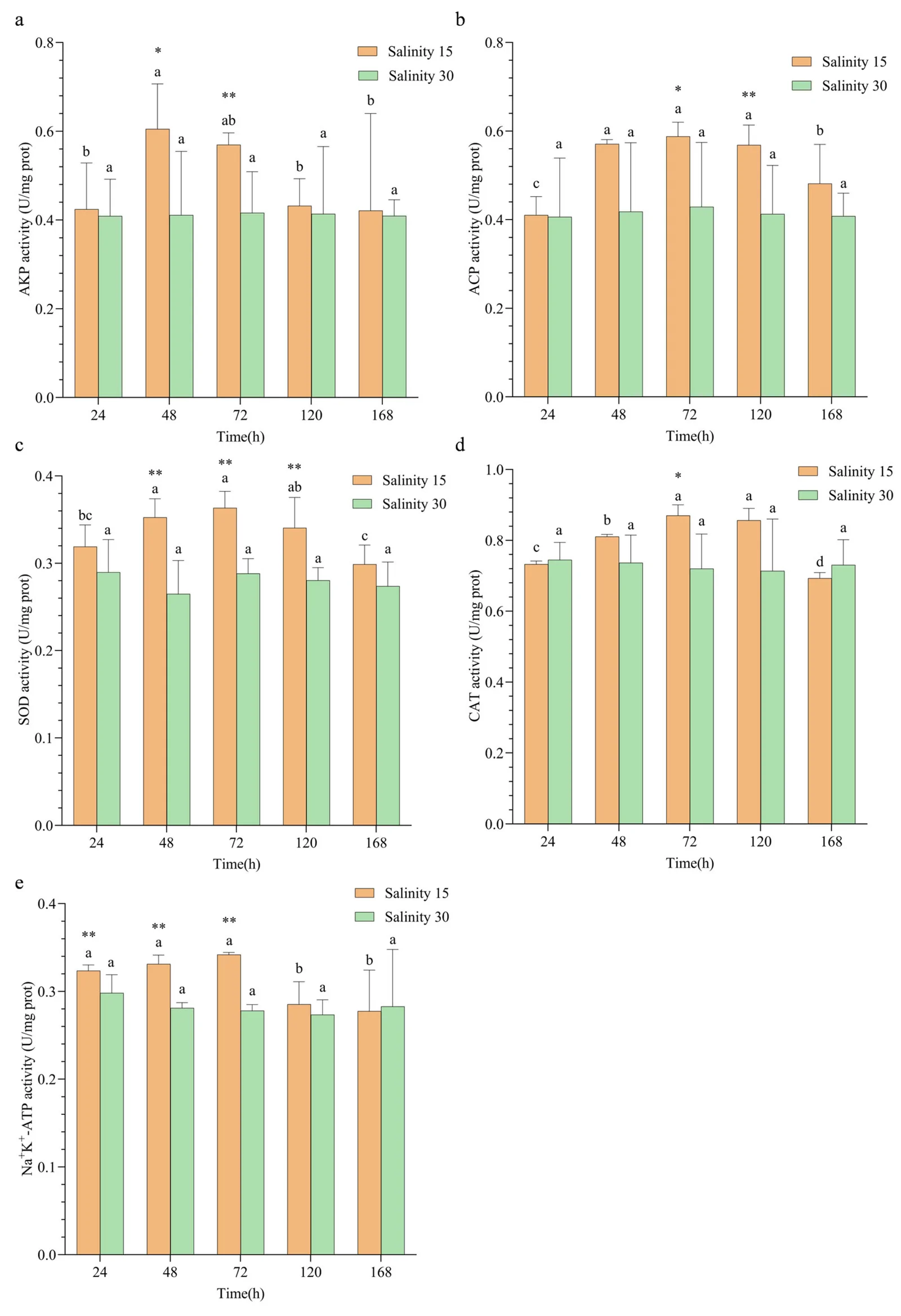
Temporal changes in the activities of immune-related, antioxidant, and osmotic regulation enzymes in the digestive gland of hard shell clams under low-salinity stress. (**a**–**e**) show the activities of AKP, ACP, SOD, CAT and NKA, respectively; * indicates significant difference compared with the control (*p* < 0.05); ** indicates highly significant difference compared with the control (*p* < 0.01). Different lowercase letters indicate significant differences among time points within the same salinity treatment (*p* < 0.05).

As shown in Figure 4, AKP activity increased at 48 and 72 h before returning to the control levels at 120 h in the 15 group. A distinct peak of 0.605 ± 0.102 U/mg prot was observed at 48 h, whereas AKP activity in the 30 group remained stable over time. Compared with the control group, AKP activity did not differ significantly at 24 h, but increased to 1.47-fold of the control level at 48 h (*p* < 0.05) and was highly significantly elevated at 72 h (*p* < 0.01). By 120 and 168 h, AKP activity had returned to the control level. No significant interaction between low-salinity stress and time was detected for AKP activity in the digestive gland (*p* > 0.05).

ACP activity in the 15 group also displayed a transient increase. Compared with the control group, ACP activity did not differ significantly at 24 h, and although it was higher at 48 h, the difference remained non-significant. At 72 h, ACP activity reached a peak of 0.588 ± 0.032 U/mg prot and was significantly higher than that in the control group (*p* < 0.05). At 120 h, ACP activity differed highly significantly from the control level (*p* < 0.01), whereas by 168 h it had returned to baseline. No significant interaction between low-salinity stress and time was observed for ACP activity (*p* > 0.05).

SOD activity in the digestive gland increased initially under low-salinity stress and then declined, reaching a peak of 0.363 ± 0.019 U/mg prot at 72 h. Compared with the control group, SOD activity in the 15 group increased slightly at 24 h, but the difference was not significant. At 48 h, however, SOD activity was significantly elevated (*p* < 0.01), reaching 1.33-fold of the control level. At 120 h, SOD activity declined slightly but remained significantly higher than that in the control group (*p* < 0.01). By 168 h, no significant difference from the control group was detected. A significant interaction between low-salinity stress and time was detected for SOD activity in the digestive gland (*p* < 0.05).

CAT activity under 15 salinity stress also showed an early increase followed by a decline, peaking at 0.870 ± 0.031 U/mg prot at 72 h. Compared with the control group, CAT activity did not differ significantly at 24 h. Although CAT activity was higher at 48 and 120 h, the differences were not statistically significant. By 168 h, CAT activity had returned to the control level. The interaction between low-salinity stress and time had a highly significant effect on CAT activity in the digestive gland (*p* < 0.01).

NKA activity in the 15 group increased initially and then gradually returned to the control level, reaching a peak of 0.342 ± 0.002 U/mg prot at 72 h after exposure. Compared with the control group, NKA activity was significantly elevated at 24 h (*p* < 0.01) and highly significantly elevated at 48 h (*p* < 0.01). At 120 and 168 h, NKA activity had returned to the control level. A significant interaction between low-salinity stress and time was detected for NKA activity in the digestive gland (*p* < 0.05).

## 4. Discussion

The survival of hard clams differed markedly among salinity regimes, indicating that *M. mercenaria* is highly euryhaline, though still physiologically challenged by salinity extremes [19]. After 168 h of exposure, survival remained 64% at salinity 50 and 52% at salinity 15, with mortality continuing to increase under both conditions. By contrast, survival remained relatively high in the salinity 20, 30, 40, and 45 groups. Additionally, preliminary experiments further suggested the broad salinity tolerance of this species, as a small number of individuals survived for 10 days at salinity 60 and for more than 7 days at salinity 10. These findings align with existing literature showing high survival in *M. mercenaria* under long-term salinity stress. Specifically, 3-month exposure to salinity 15, 25, and 35 induced zero mortality; however, hyposaline exposure (salinity 15) significantly suppressed growth, whereas elevated salinity (salinity 35) supported sustained growth [8,20]. Under 21-day acute stress, hard clams tolerate a salinity range of salinity 5–35, whereas extreme hypersalinity (salinity 45) causes total mortality within 7 days, highlighting a narrower safety margin for hypersaline conditions. This asymmetric tolerance is partially driven by behavioral strategies: under hyposaline stress, clams reduce filtration rates and close their valves to delay hemolymph osmotic equilibration, whereas hypersaline acclimation involves rapid osmotic adjustment within 12 h [18]. At the molecular level, transcriptomic responses exhibit pronounced tissue- and age-dependent specificity. In long-term stressed gills, SLC6 family amino acid transporter genes responsible for osmolyte synthesis are predominantly upregulated under hypersaline conditions [8]. Conversely, in acutely stressed hemocytes, homologous solute transporter genes display completely opposing regulatory patterns between hypo- and hyper-salinity [18]. Short-term low-salinity exposure also triggers extensive transcriptomic remodeling, with distinct response dynamics between adult and juvenile clams [17]. Taken together, these observations indicate that adult hard clams rely on behavioral buffering (e.g., valve closure) to survive moderate hyposaline stress, albeit at the physiological cost of growth inhibition and immune alteration. Conversely, while moderate hypersalinity promotes growth, extreme hypersalinity poses a far higher risk of acute mortality.

The gills of hard clams are central organs for respiration, filtration, and ion exchange and therefore represent one of the first tissues to respond to salinity challenge [21]. In the present study, gill structure remained intact and well organized at salinity 30, indicating that this salinity fell within the normal physiological range for the tested animals. Under both hypersaline (50) and hyposaline (15) treatments, however, early phase exposure (within 72 h) caused pronounced filament shrinkage and disorganization, whereas prolonged exposure (after 72 h) resulted in increased inter-filament spacing, nuclear displacement, and cellular degeneration. These findings suggest that hard clams can initially withstand abrupt salinity shifts, but sustained exposure progressively compromises gill integrity and may impair respiratory and osmoregulatory function. Similar histopathological responses have been reported in other bivalves. Low-salinity stress in Solen dactylus causes abnormal hyperplasia, necrosis, and atrophy in gill filaments [22], whereas *Crassostrea iredalei* exhibits gill necrosis and structural dissociation under low-salinity conditions [23]. Likewise, salinity shifts induce marked physiological and transcriptomic responses in the gill of *Solenaia oleivora* (notably, this species is a freshwater mussel), including structural injury and osmoregulatory disturbance [24]. At the molecular level, salinity stress has been shown to reshape gill metabolism and transcriptional regulation, particularly pathways related to cytoskeletal remodeling, immune signaling, ion transport, apoptosis, and antioxidant defense [25]. Acute hyposaline shock can induce sustained swelling of bivalve gill cells; nevertheless, the capacity for whole-organ volume compensation in gill tissue appears limited during abrupt salinity shifts [26]. Our results therefore are consistent with the view that gill injury is not merely a passive consequence of osmotic disturbance, but part of a broader stress-response network involving structural remodeling, physiological defense, and metabolic adjustment.

As key hydrolases, AKP and ACP play important roles in non-specific immune defense. They participate in the clearance of exogenous substances and are associated with the phagocytic activity of hemocytes. Specifically, ACP is a typical lysosomal enzyme involved in phagocytosis and intracellular digestion, whereas AKP hydrolyzes phosphate esters and is associated with immune-related physiological regulation [27,28]. Salinity stress alters hemolymph osmotic status and thereby modulates immune indices in shellfish. Previous studies have shown that ACP and AKP activities in *Solenaia oleivora* increase during the early phase of salinity stress, whereas ACP activity declines after 48 h [24]. Under short-term low-salinity stress, AKP and ACP activities are also significantly enhanced in *Crassostrea nippona* [12]. These responses typically follow a pattern of early elevation followed by decline or stabilization. In the present study, ACP and AKP activities in the digestive gland of hard clams under both high- and low-salinity stress followed a similar temporal pattern of early activation followed by recovery toward the control level, consistent with previous reports. Recent work in hard clams has also shown that salinity stress alters hemocyte immune traits and immune-related transcriptional pathways [18]. The transient elevation of ACP and AKP observed here therefore may reflect an early compensatory activation of nonspecific immune defense, whereas the subsequent decline toward baseline suggests either partial acclimation or attenuation of immune activation under prolonged stress.

Environmental stress can induce excessive production of reactive oxygen species (ROS), including hydrogen peroxide, superoxide anions, and hydroxyl radicals, which may lead to oxidative damage, metabolic disruption, and even mortality [29]. To counteract ROS-mediated injury, organisms have evolved antioxidant defense systems composed of enzymatic and non-enzymatic components [30]. Among these, SOD is a core antioxidant enzyme that catalyzes the dismutation of superoxide radicals into hydrogen peroxide, thereby protecting cells and maintaining redox homeostasis [31,32]. CAT, a marker enzyme of peroxisomes, further decomposes hydrogen peroxide into water and oxygen, preventing the formation of highly reactive hydroxyl radicals and, together with SOD, constituting a major antioxidant defense mechanism [33]. Numerous studies have demonstrated salinity-induced changes in SOD and CAT activities in aquatic invertebrates. For example, when *Apostichopus japonicus* is transferred from salinity 30 to salinity 35, SOD activity in coelomic fluid rises significantly within 3 h and then gradually returns to the control level [30]. In *Mytilus galloprovincialis*, reduced salinity decreases gill SOD and CAT activities, whereas further salinity increase is associated with recovery of these enzyme activities [34]. Under acute low-salinity stress, SOD and CAT activities in Chlamys nobilis peak at 6 h and decline significantly by 24 h [35]. In the present study, both SOD and CAT activities in hard clams displayed a characteristic pattern of initial elevation followed by decline under both high- and low-salinity stress, broadly consistent with previous reports. These findings suggest that hard clams initially mobilize antioxidant defenses to counteract ROS accumulation, whereas prolonged salinity stress may suppress antioxidant capacity or increase oxidative burden beyond the compensatory range of the enzymatic system. This interpretation is also consistent with recent salinity studies in bivalves showing that the recovery of antioxidant capacity depends strongly on the intensity and duration of exposure [36].

As a transmembrane ion-transporting protein, Na+/K+-ATPase (NKA) drives active ion transport across cell membranes and plays a fundamental role in osmotic regulation in mollusks and other aquatic animals [37]. While the gills serve as the primary interface for ion exchange, the digestive gland plays a crucial secondary role in bivalve osmo-ionic homeostasis through high NKA activity, supporting intracellular ion balance and metabolic partitioning during salinity stress [38]. Here, assessing digestive gland NKA activity provides a systemic perspective that complements gill histopathology, helping to link localized structural injury in the gills to whole-animal physiological impairment. In contrast to a simple decline, the present results showed that NKA activity in the digestive gland increased during the early phase of both high- and low-salinity exposure and then gradually returned toward the control level as exposure continued. This pattern suggests that NKA is involved in short-term osmotic adjustment in hard clams. When external osmotic pressure changes, hard clams rapidly activate regulatory mechanisms to maintain ion homeostasis, and changes in NKA activity may reflect this adaptive adjustment. Similar salinity-responsive changes in NKA activity have been reported in other shellfish. In *Pinctada fucata*, NKA activity decreases only after 24 h of low salinity, whereas it is significantly elevated after 12 h of low salinity and after 12–24 h of high salinity [39]. In *Solenaia oleivora*, NKA activity decreases in the gill, hepatopancreas, and foot during 3–48 h of acute high-salinity stress and also declines in the adductor muscle during 6–48 h of salinity stress [40]. More recent integrative studies further suggest that NKA regulation is tightly coupled to broader metabolic reorganization, including amino acid metabolism, oxidative balance, and energy allocation in salinity-stressed bivalves [8,17]. Accordingly, the present results are consistent with the view that NKA is a key component of the compensatory response to osmotic disturbance in hard clams, particularly during the early phase of exposure, whereas the later return toward baseline may indicate either successful acclimation or reduced regulatory capacity under sustained stress.

Overall, the present study suggests that hard clams possess substantial physiological plasticity under salinity challenge, but that this plasticity depends on coordinated regulation of tissue integrity, immune defense, antioxidant capacity, and ion transport. Recent studies in *M. mercenaria* have increasingly emphasized transcriptomic, hemocyte-based, and biochemical responses to salinity stress; our findings extend this framework by relating these responses to progressive gill injury and time-dependent enzymatic regulation [8,17,18]. Together, these results suggest that hard clams tolerate short-term salinity fluctuation through compensatory adjustment, whereas prolonged exposure may either transition from acclimation to progressive functional impairment, or maintain stable acclimated status depending on salinity magnitude. This distinction is likely to be particularly relevant for pond and estuarine aquaculture, where recurrent salinity fluctuations associated with extreme weather events and altered freshwater input are becoming increasingly common.

## 5. Conclusions

This study shows that *Mercenaria mercenaria* can tolerate substantial salinity fluctuations, with greater tolerance to hypersaline conditions than to extreme hypersalinity. Salinity stress caused progressive gill injury and induced coordinated changes in immune-, antioxidant-, and osmoregulatory enzyme activities, indicating that salinity adaptation in hard clams relies on an integrated physiological response. These findings provide a physiological basis for salinity management in aquaculture and for the selective breeding of hard clam strains with improved resilience to fluctuating salinity.

## Data Availability

The original contributions presented in this study are included in the article. Further inquiries can be directed to the corresponding author.
